# The Emerging Role of Cell Transdifferentiation in Skeletal Development and Diseases

**DOI:** 10.3390/ijms23115974

**Published:** 2022-05-26

**Authors:** Ke Wang, Chi Ma, Jian Q. Feng, Yan Jing

**Affiliations:** 1 Department of Biomedical Sciences, Texas A&M University College of Dentistry, Dallas, TX 75246, USA; kewang79@gmail.com (K.W.); fengjianquan888@gmail.com (J.Q.F.); 2 Division of Orthodontics, University of Connecticut Health Center, Farmington, CT 06030, USA; 3 Center for Excellence in Hip, Scottish Rite for Children, Dallas, TX 75219, USA; chi.ma@utsouthwestern.edu; 4 Department of Orthopaedic Surgery, UT Southwestern Medical Center, Dallas, TX 75390, USA; 5 Department of Orthodontics, Texas A&M University College of Dentistry, Dallas, TX 75246, USA

**Keywords:** chondrocyte, tendon cells, endochondral ossification, osteogenesis, cell transdifferentiation

## Abstract

The vertebrate musculoskeletal system is known to be formed by mesenchymal stem cells condensing into tissue elements, which then differentiate into cartilage, bone, tendon/ligament, and muscle cells. These lineage-committed cells mature into end-stage differentiated cells, like hypertrophic chondrocytes and osteocytes, which are expected to expire and to be replaced by newly differentiated cells arising from the same lineage pathway. However, there is emerging evidence of the role of cell transdifferentiation in bone development and disease. Although the concept of cell transdifferentiation is not new, a breakthrough in cell lineage tracing allowed scientists to trace cell fates in vivo. Using this powerful tool, new theories have been established: (1) hypertrophic chondrocytes can transdifferentiate into bone cells during endochondral bone formation, fracture repair, and some bone diseases, and (2) tendon cells, beyond their conventional role in joint movement, directly participate in normal bone and cartilage formation, and ectopic ossification. The goal of this review is to obtain a better understanding of the key roles of cell transdifferentiation in skeletal development and diseases. We will first review the transdifferentiation of chondrocytes to bone cells during endochondral bone formation. Specifically, we will include the history of the debate on the fate of chondrocytes during bone formation, the key findings obtained in recent years on the critical factors and molecules that regulate this cell fate change, and the role of chondrocyte transdifferentiation in skeletal trauma and diseases. In addition, we will also summarize the latest discoveries on the novel roles of tendon cells and adipocytes on skeletal formation and diseases.

## 1. Introduction

The human musculoskeletal system is composed of bones, muscles, cartilage, tendons, and ligaments, which are all derived from mesenchymal stem cells (MSC) in the mesoderm. Proliferating MSC enter a lineage following their commitment to that particular pathway. These lineage-committed cells progress through several transitory stages and undergo a maturation stage. The end-stage differentiated cells, like hypertrophic chondrocytes (HC) and osteocytes, are expected to expire and to be replaced by newly differentiated cells arising from the same lineage pathway [1].

**Figure 1:**
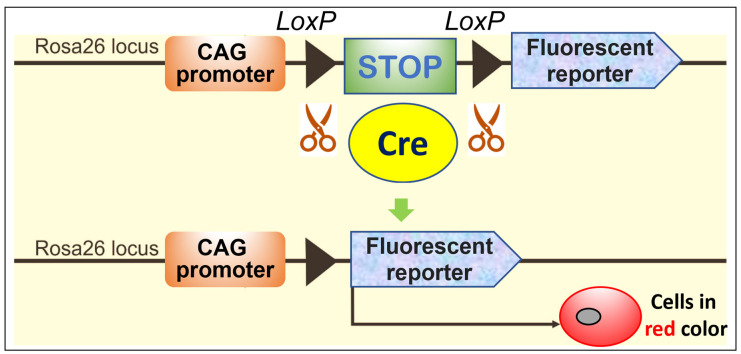
The mechanism of cell lineage tracing. The Cre excises the STOP sequence, and it activates the tomato reporter in the specific cell line when the mouse has both the Cre and loxP expression. As a result, this specific cell lineage is labeled in red color.

Cell transdifferentiation from one type of mature cell to another occurs in many processes, including gastrulation, neural crest and somite dissociation, craniofacial development, wound healing, organ fibrosis, and tumor metastasis [2,3]. It has long been proposed that chondrocytes, tendon cells, and adipocytes can change their identity, but the phenomena were primarily observed in vitro. Recently, remarkable progress in imaging and cell lineage tracing technologies has shed new light on the secrets of skeletal biology. In particular, cell lineage tracing provides a rigorous way to study cell fate in vivo [4,5]. Briefly speaking, a recombinase enzyme only expressed in a specific type of cell will stimulate the expression of a reporter gene. This cell type and their descendants will then become permanently labeled. The Cre-loxP system is commonly used in lineage tracing. Cre (the recombinase enzyme) will excise the STOP sequence between two *loxP* sites and activate the reporter in a specific cell line (Figure 1). In some cases, the investigator can choose a favorable time point to activate Cre by using a drug, such as tamoxifen, causing Cre to fuse to a modified estrogen receptor (Cre^ERT2^) [4]. Fluorescent reporters have become the standard lineage tracing approach because they dramatically reduce the complexity and improve the accuracy and the efficiency of cell fate tracing. Another commonly used reporter is the *E. coli* lacZ gene, which encodes the protein beta-galactosidase [6]. This enzyme causes bacteria expressing the gene to appear blue when grown on a medium that contains the substrate analog X-gal.

In this paper, we will first review the transdifferentiation of chondrocytes to bone cells during endochondral bone formation. Specifically, we will include the history of the debate on chondrocyte fate during bone formation, the key findings obtained in recent years on critical factors and molecules that regulate this cell fate change, and the role of chondrocyte transdifferentiation in skeletal trauma and diseases. In addition, we will also summarize the latest discoveries on the novel roles of tendon cells and adipocytes in skeletal formation and diseases.

## 2. Chondrocytes Directly Transdifferentiate into Bone Cells during Endochondral Bone Formation

Most bones (80%) in mammals are formed through endochondrogenesis. In embryonic development, endochondrogenesis starts from the condensation of mesenchymal cells which then differentiate into chondrocytes, followed by pre-hypertrophic and HC. These cells secrete unique matrix proteins and calcify cartilage matrices. Subsequently, the terminally differentiated HC were thought to undergo degeneration and apoptosis. This induces the invasion of osteoclasts and osteoblast precursor cells from underlying bone marrow and vasculature to remove dead chondrocytes and deposit new bone, respectively [7,8].

Although the theory of chondrocyte cell death and cartilage replacement by bone has been dominant in the field for many decades, there has always been a dispute over the fate of HC. Strelzoff first reported cartilage resorption and osteogenesis in chick embryos in 1873 [9], proposing the “replacement” theory which later became well-accepted. In the following decades, however, multiple researchers observed indications of a change in cell fate from chondroblasts into bone-like cells in the embryonic and the postnatal long bones of chicks [10,11,12,13]. Furthermore, a similar phenomenon was documented in pig long bones by Carey [14]. These studies established the main premise that cartilage is very unstable, and at least some of these cells will directly give rise to bone cells rather than undergo programmed cell death. However, the data consisted of largely phenotypic descriptions and cannot serve as conclusive evidence for the transition from chondrocytes to osteogenic cells.

### 2.1. Emerging Evidence of Chondrocyte Transdifferentiation Using Cell Lineage Tracing

In the past decade, cell lineage tracing started to become a rigorous and a reliable method to trace cell fate in vivo. With non-inducible Col10a1-Cre, which specifically labels HC and their descendants, researchers found that not only the HC in the growth plate were labeled, but also osteogenic cells in the primary ossification centers of fetal, neonatal and adult bone [15,16,17]. These cells were located near the chondro-osseous junction, throughout the trabeculae surfaces, in the endosteum, and embedded within the cortical bone matrix. Co-expression of bone markers confirmed that the descendants of HC become Col1a1^+^ or Osterix^+^ osteoblasts, and Sclerostin^+^ osteocytes [15,16,17]. The osteoblasts derived from Col10a1-expressing HC represent approximately 70% of all mature osteoblasts in the endochondral bones of one-month-old mice [15]. Notably, at no stage were these Col10a1 lineage cells found in the perichondrium/periosteum, thereby confirming the HC origin of these bone cells. These findings were further corroborated with tamoxifen-induced Col10a1-Cre^ERT2^; LacZ mice [16]. When tamoxifen was administered at E13.5, before primary ossification center formation in the long bone, approximately 80% of Col10a1-Cre^ERT2^; LacZ^+^ cells in the bone expressed Col1a1 (Type I Collagen, mainly expressed by osteoblast lineage) at both E18.5 and P5, suggesting that most HC-derived cells become osteoblasts [16]. The same phenomenon was also observed during secondary ossification, where HC labeled by Col10a1-Cre changed their cell fate into osteoblasts in the epiphysis at 3 weeks of age [15].

**Figure 2:**
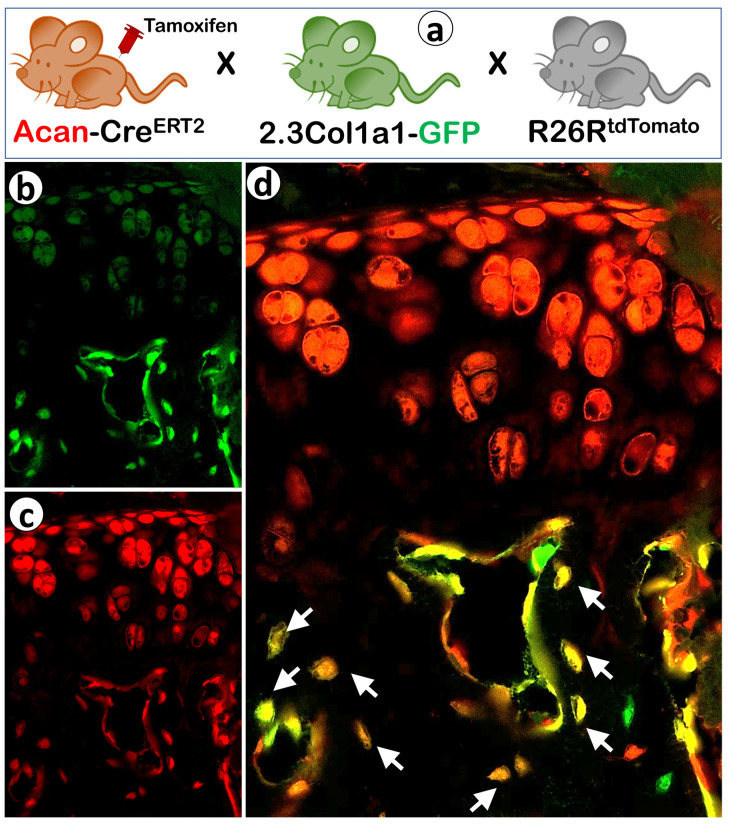
Cellular co-localization of the chondrocyte-derived tomato marker and a 2.3 Col1a1-GFP osteoblast-specific marker in the TMJ condyle cartilage and underlying trabecular bone. (**a**) The schematic diagram illustrates the generation of triple mice containing Acan-Cre^ERT2^, 2.3Col1a1-GFP, and R26R^tdTomato^ along with a tamoxifen induction to activate the Cre event. (**d**) Confocal images of the GFP (**b**) and Tomato (**c**) signals in the articular cartilage of a 6-week-old mouse. (**c**) The merged image of GFP and Tomato signals, which revealed non-chondrocyte-derived bone cells (green), chondrocyte-derived bone cells with no GFP activation yet (red), and chondrocyte-derived bone cells expressing type I collagen (yellow as denoted with arrows).

In addition to long bones, the transdifferentiation of chondrocytes to bone cells is also identified in other skeletal components like the mandibular condyle. By crossing Acan-Cre^ERT2^; R26R^tdTomato^ (induced at P14) mice with the 2.3 Col1a1-GFP line [18] (a marker for osteoblasts and osteocytes), Jing et al. observed a large portion of chondrocyte-derived Tomato^+^ cells co-expressing GFP in the condylar subchondral bone [10] (Figure 2). To quantify the contribution of chondrocyte-derived osteogenesis during condyle formation, they used non-inducible Col10a1-Cre so that the HC-derived bone cells were automatically traced. By postnatal day 21, the percentage of chondrocyte-derived bone cells in the superior, middle, and inferior regions of the subchondral bone was 80%, 70%, and 40%, respectively [10], which is consistent with the long bone studies [16,19]. The cranial base is another bone region formed through endochondral ossification [20]. Sakagami et al. investigated the cellular fates of Col2a1-expressing cells in the craniofacial skeletal complex using Col2a1-Cre; R26R^tdTomato^; 2.3 Col1a1-GFP mice [21]. As expected, the majority of skeletal cells in the cranial base were marked by the Tomato signal.

Chondrocyte transdifferentiation can occur rapidly [22,23]. When tamoxifen was administered to Col10a1-Cre^ERT2^; LacZ mice at E14.5 just before primary ossification in the long bones, all the LacZ^+^ cells were HC by 24 h. However, 36 h after tamoxifen induction, some LacZ^+^ cells already expressed Col1a1 and Osterix [16]. This evidence shows that transdifferentiation from embryonic HC to osteoblasts could take as little as 12 h [16]. Similarly, Zhou et al. administered tamoxifen to pregnant females with Acan-Cre^ERT2^; LacZ embryos at age E15.5 and found weak LacZ signals in both chondrocytes and HC 9 h later. At 18 h, many LacZ^+^ chondrocytes and HCs along with very few LacZ^+^ bone cells in the primary spongiosa were detected. By 24 h post-injection, significantly more bone cells in the primary spongiosa were positive for LacZ [15]. Chondrocyte transdifferentiation also occurs fast in the postnatal stage. When tamoxifen was administrated in Col10a1-Cre^ERT2^; LacZ mice at P5, LacZ^+^ Col1a1^+^ cells were found 24 h post-injection [16]. A separate study by Jing et al. using Acan-Cre^ERT2^; R26R^tdTomato^ pups also revealed Tomato^+^ osteocytes in the TMJ condylar bone 24 h after tamoxifen injection [10].

Taken together, based on the new findings of chondrocyte transdifferentiation discovered via cell lineage tracing, a new theory is proposed: chondrogenesis and osteogenesis are two sequential phases in a continuous lineage-defined process during endochondral bone formation [24]. Chondrocyte transdifferentiation connects these two phases and precisely secures the intrinsic transmission of the skeletal template message from cartilage to bone. Several genes participate in the regulation of chondrocyte transdifferentiation.

### 2.2. Signals That Are Critical to Chondrocyte Transdifferentiation

#### 2.2.1. Diverse Roles of BMP Signaling in Chondrocyte Transdifferentiation

BMP (Bone Morphogenetic Proteins) signaling is vital for endochondral bone formation [25,26,27,28,29,30]. It is directly involved in several debilitating skeletal pathologies including fibrodysplasia ossificans progressiva, Marfan syndrome, and osteoarthritis [31]. Deletion of *Bmp2* and *Bmp4* genes or *Bmp2* gene alone in chondrocytes using Col2a1-Cre^ERT2^ (tamoxifen given at E12.5) mice resulted in severe defects in chondrocyte proliferation, differentiation, and apoptosis in growth plate, and also delayed formation of the primary ossification center in E14.5 and E18.5 embryos [29]. *Bmpr1a* and *Bmpr1b* are two critical type I receptors expressed throughout the growth plate. The ablation of these receptors revealed not only severe chondrodysplasia but also the virtual absence of endochondral ossification during embryonic development [26,32,33]. Interestingly, when removing Bmpr1a in chondrocytes during postnatal growth, opposite changes were found in the metaphysis and epiphysis: an arrest of long bone growth with no sign of a metaphysis and a massive increase in epiphysis mass [34,35].

Taking advantage of cell lineage tracing, Jing et al. generated a compound mouse line consisting of Acan-Cre^ERT2^; R26R^tdTomato^; Bmpr1a^fx/fx^ with a one-time tamoxifen injection at postnatal day 3 [24]. At 2 months old, these Bmpr1a conditional knockout (cKO) mice displayed a complete arrest in chondrocyte transdifferentiation from the growth plate, resulting in the lack of a metaphysis, whereas numerous Tomato^+^ bone cells were found in the control metaphysis. On the other hand, the epiphysis of cKO mice was largely expanded and malformed along with increased Tomato^+^ chondrocytes and chondrocyte-derived red bone cells compared to the controls. This evidence demonstrates that: (1) *Bmpr1a* plays an inhibitory role in epiphysis formation but a stimulatory role in metaphysis formation, and (2) *Bmpr1a* directly controls cell transdifferentiation regardless of its inhibitory or stimulatory role in articular cartilage or in growth plates [24].

On the other hand, conditionally removed *Bmpr1a* in bone cells only using 3.6 Col 1a1-Cre [24] displayed no apparent change in bone length or shape except an increase in bone volume. Similar phenotypes were also found in the deletions of *Bmpr1a* using 3.2 Col 1-Cre [28,30] or Dmp1-Cre [36]. Together, the studies on chondrocytes and bone cells using *Bmpr1a* cKO lines with a cell tracing background precisely confirmed that chondrogenesis and osteogenesis are a part of one continuous developmental and lineage-defined biological process. BMP signaling plays a key role in the coupling of these two phases via regulation of chondrocyte transdifferentiation, although the manner of regulation is strikingly different depending on the region of the bone: it enhances the transdifferentiation to bone cells in the metaphysis from chondrocytes in the growth plate and slows the transdifferentiation to epiphyseal bone cells from chondrocytes in the perichondrium.

#### 2.2.2. Dual Function of Wnt/β-Catenin Signaling in Chondrocyte Cell Fate Change

Wnt/β-catenin signaling plays a critical role in regulating skeletal development and growth [37,38,39,40,41,42,43,44]. Specific ablation of β-catenin in chondrocytes using Col10a1-Cre or Col2a1-Cre^ERT2^ led to impaired terminal differentiation of chondrocytes, substantial deficiency of mature osteoblasts, altered RANKL:OPG ratio in HC, and a severe loss of subchondral bone [37,39,40,45]. Lineage tracing revealed that chondrocyte transdifferentiation in the long bones was almost completely blocked in mice lacking β-catenin activity in HC [45]. Conversely, gain of β-catenin activity in HC promoted the transdifferentiation of chondrocytes and interfered with the removal of late HC, resulting in a continuously mineralized hypertrophic core in the embryo and an osteopetrotic-like phenotype in adult mice [45]. Therefore, β-catenin has dual functions in trabecular bone homeostasis by regulating both osteoclastogenesis and osteogenesis via the transdifferentiation from chondrocytes to osteoblasts [45].

In addition to long bones, β-catenin also plays an essential role in chondrocyte transdifferentiation during mandibular condylar growth [46]. Deletion of β-catenin with either Acan-Cre^ERT2^ or Col10a1-Cre resulted in a large reduction of chondrocyte-derived bone cells, severe defects in cell proliferation and differentiation in both chondrocytes and bone cells, along with a significant decrease in subchondral bone volume during mandibular condylar growth. In contrast, constitutive activation of β-catenin in chondrocytes using Acan-Cre^ERT2^ promoted both chondrocyte proliferation and transdifferentiation into bone cells, resulting in an increased volume of poorly formed immature subchondral bone [46].

#### 2.2.3. Vital Role of Runx2 in Chondrocyte Transdifferentiation

Runx2 (runt-related transcription factor 2) is a transcription factor that belongs to the Runx family, which is composed of Runx1, Runx2, and Runx3 [47]. Runx2 is essential for osteoblast differentiation and chondrocyte maturation. Loss-of-function mutations in human RUNX2 are responsible for cleidocranial dysplasia, a rare autosomal-dominant disorder characterized by skeletal and dental abnormalities including large fontanelles, hypoplasia or the absence of clavicles, supernumerary teeth, and short stature [48]. When deleting *Runx2* in HCs using Col10a1-Cre mice, the apoptotic activity of HC was increased and their transdifferentiation was interrupted, resulting in a lack of primary spongiosa and chondrocyte-derived osteoblasts at E16.5. However, the bone structure, and volume, and all bone histomophometric parameters were similar between *Runx2* KO and control mice at 6 weeks of age [49]. These findings indicate that Runx2 expression is required for chondrocyte survival and transdifferentiation in embryonic and neonatal stages, but not for acquiring normal bone structure and volume in pediatric and adult mice.

#### 2.2.4. The Importance of Sox9 in Chondrocyte Maturation and Transdifferentiation

Sox9 (sex determining region Y-box 9) is an essential transcriptional factor for chondrogenesis. It is expressed in chondroprogenitor cells [50] to secure chondrocyte lineage commitment, promote cell survival, and transcriptionally activate the genes for many cartilage-specific structural components and regulatory factors [51]. *Sox9* activates Col10a1 transcription in HC by binding to its promoter cooperatively with myocyte enhancer factor 2C (Mef2c) [52]. *Sox9* null mice displayed reduced chondrocyte hypertrophy associated with Col10a1 expression in the hypertrophic zone [52,53]. Furthermore, persistent Sox9 expression in the growth plate caused an inhibition of chondrocyte transdifferentiation to bone cells in trabecular bone, with a decreased expression of *MMP9*, *MMP13*, Sp7, and *Col1a1* [54]. Another recent study conditionally deleted Sox9 in the chondrocyte lineage using Acan-Cre^ERT2^ line, followed by single cell RNA-sequencing analysis of chondrocytes extracted from control and mutant growth plates and articular cartilage [55]. They concluded that the chondrocytes from both regions exhibit osteogenic plasticity throughout life, and *Sox9* prevents their premature or ectopic osteoblastogenesis. These studies demonstrate that *Sox9* plays a key role in controlling chondrocyte maturation and transdifferentiation.

#### 2.2.5. Critical Regulation of Ihh Pathway in Coupling Chondrogenesis and Osteogenesis

Indian hedgehog (Ihh) is expressed by prehypertrophic and HC, and it plays a critical role in regulating endochondral bone formation [56,57]. Prior studies have revealed a direct link between hedgehog signaling and the development of OA and heterotopic ossification [58]. Like all Hedgehog (Hh) proteins, Ihh signals via the seven-pass transmembrane protein Smoothened (Smo) to modulate gene expression [56]. Conditional removal of Ihh from chondrocytes in newborn Col2a1-Cre^ERT2^; Ihh^flox/flox^ mice resulted in a dwarfism phenotype due to a loss of columnar structure in the growth plate and continuous loss of trabecular bone [59]. Similarly, ablation of Smo in chondrocytes led to a 50% reduction in chondrocyte proliferation [60], prevented formation of a normal bone collar, and abolished development of the primary spongiosa [61]. Thyroid hormone (TH) increases the transcription levels of Ihh via THRβ1, thereby promoting chondrocyte hypertrophy and transdifferentiation during secondary ossification [22,62,63]. TH deficient mice had a lack of secondary ossification or defective epiphyseal bone formation [22,63], which could be rescued by TH treatment for 10 days [63]. These data provide evidence that chondrocyte to osteoblast transdifferentiation is TH dependent. Investigators in another study found a great reduction in callus formation and bone mineralization after fracture in Ihh^−/−^ zebrafish, while chondrocyte proliferation in the fracture region remained unaffected [64]. In addition, the deletion of *Bmpr1a* using Acan-Cre^ERT2^ leads to a dramatic decrease in Ihh expression in the growth plate [34]. There is also evidence to show that Ihh controls cell transdifferentiation from chondrocytes to bone cells via an activation of the Wnt signaling pathway [59]. Taken together, these studies strongly suggest that the Ihh pathway couples chondrogenesis and osteogenesis.

### 2.3. The Role of Chondrocyte-Derived Osteogenesis in Skeletal Pathological Conditions

#### 2.3.1. Bone Fracture

Numerous studies have proven the key contribution of chondrocyte transdifferentiation in the fracture healing of long bones and the mandible [15,65,66,67], using cell lineage tracing. Zhou et al. administered a tamoxifen injection to 2- to 3-month-old Acan-Cre^ERT2^; 2.3 Col1a1-GFP; R26R^tdTomato^ mice 6 days after tibia fracture, a time prior to chondrocyte differentiation [15]. Some Tomato^+^ chondrocytes were then identified in the cartilage callus 9 days after the fracture. Fourteen days after the fracture, almost all cells in the callus, including both cartilage and bone regions, were positive for Tomato signal. Many of these cells also expressed 2.3 Col1a1-GFP, indicating that chondrocytes present in the callus became Col1a1-expressing bone cells. Ossification was almost complete in the repaired callus 29 days post-fracture and the number of Tomato^+^ GFP^+^ cells substantially increased compared to the day-14 callus [15]. Similar events have been observed when using HC grafts to heal tibial defects [16,68] or mandibular fractures [69]. Furthermore, Hu et al. [67] showed a spatially dependent phenotypic overlap between HC and osteoblasts at the chondro-osseous border within the fracture callus, in a region defined as the transition zone. HC in this zone also actively expressed pluripotency factors such as Sox2, Oct4 (Pou5f1), and Nanog. Conditional knockout of Sox2 resulted in the reduction of the fracture callus and a delay in the conversion of cartilage to bone.

#### 2.3.2. Osteoarthritis

Osteoarthritis (OA) is characterized by a progressive degeneration of the articular cartilage, as well as pathological changes to the surrounding tissues of joints such as subchondral bone, synovial membrane, and ligaments [70,71,72]. No therapy is currently available to completely prevent OA initiation or progression due to limited understanding of its pathological mechanisms. Some recent studies have indicated a likely role of altered chondrocyte transdifferentiation in OA development. Ji et al. performed scRNA-seq analysis on 1464 chondrocytes from 10 patients with OA undergoing knee arthroplasty surgery and found two subpopulations of HC in human OA cartilage [73]. One cluster expressed unique markers that were enriched for genes related to cartilage development, whereas the other was enriched for genes related to ossification and mineralization. Ruscitto et al. surgically induced OA on the TMJ of miniature pigs using a disc perforation model, and they observed increased Col2a1^+^ BSP^+^ or Col2a1^+^ Runx2^+^ cells in the condyle cartilage, indicating a putative transdifferentiation from chondrocyte to bone cells [74]. Roelofs et al. found Col2a1^+^ cells in the forming osteophyte which co-expressed osteoblast markers like Ocn and Col1a1. They also confirmed that Sox9^+^ lineage was one source of these hybrid cells, which co-expressed Col2a1 and Col1a1 mRNA. These findings indicate that the early osteophyte contains Sox9-derived hybrid skeletal cells [75]. In addition, there are other studies indicating that Sox9 is required to maintain the integrity of articular cartilage and inhibit chondrocyte osteoblastogenesis under loading stimulation [55,76].

#### 2.3.3. Hypophosphatemic Rickets

Dentin matrix protein 1 (DMP1), a non-collagenous phosphoprotein that belongs to the small integrin binding ligand N-linked glycoproteins (SIBLING) family, is essential for normal postnatal chondrogenesis and osteogenesis [77,78,79,80]. Mutation of DMP1 in human [80,81] and deletion of *DMP1* in mice [79,80] and rabbits [82] lead to defects in osteocyte maturation and an increase of fibroblast growth factor 23 (FGF23), a key factor for autosomal recessive hypophosphatemic rickets type I [83,84,85]. For a long time, hypophosphatemic rickets have been thought to be caused by an inhibition of chondrogenesis, leading to an accumulation of HC and a failure to replace cartilage by bone [86]. To precisely investigate the mechanisms by which DMP1 and phosphorus regulate chondrogenesis and osteogenesis, Li et al. deleted *DMP1* in the background of Acan-Cre^ERT2^; R26R^tdTomato^ or Col10a1-Cre; R26R^tdTomato^ [87]. Both tracing lines displayed an acceleration of chondrogenesis and transdifferentiation in the mandibular condyles of *Dmp1* null mice compared to the control mice. This phenotype can be restored by administering neutralizing FGF23 antibodies or a high phosphorus diet. Further studies into the mechanism revealed that the hypophosphatemia-induced acceleration of chondrocyte transdifferentiation was caused by an increase in β-catenin expression [87]. Together, the cell lineage tracing study revised our understanding on hypophosphatemic rickets, which indeed starts from an acceleration of chondrogenesis, leading to more chondrocyte-derived bone cells. Due to a decrease in phosphorus caused by the removal of *Dmp1*, the chondrocyte-derived bone cells are immature in cell differentiation, thereby instigating a defect in mineralization [87].

In summary, recent studies using cell lineage tracing have demonstrated that chondrocytes play a key role during endochondrogenesis in both normal and skeletal disease states. The focus of future studies should be shifted away from the bone marrow and instead be directed towards developing novel methods to control chondrocyte transdifferentiation. New avenues of research will become available as we improve our understanding of this phenomenon, such as the development of regenerative therapies for skeletal trauma and diseases by regulating the aforementioned critical factors involved in chondrocyte transdifferentiation.

## 3. Beyond Their Conventional Role in Joint Movement, Tendon Cells Directly Participate in Skeletal Development and Diseases

Tendon is a tough, high-tensile-strength band of dense fibrous tissue that connects muscle to bone. Ligament is histologically similar to tendon and connects one bone to another. The sole function of tendon/ligament (simply called tendon in this review) was thought to be the transmission of muscle forces for joint stabilization. However, like the debate on the fate of chondrocytes, there was indirect evidence indicating that tendon may play a role in skeletal formation. Summers et al. found that tendon forms a fibrocartilaginous pad followed by sesamoid bone formation under comprehensive loading in fish [88]. Furthermore, tendon calcifies in birds under tensile loadings [89,90]. In humans, calcific tendonitis occurs either through degenerative or reactive calcification [91,92,93,94] and skeletal diseases like osteophyte formation [95,96,97] always occur in tendon insertion sites.

Scleraxis (Scx), a transcriptional factor highly expressed in tendon, is indispensable for tendon cell differentiation and maturation [94,98,99,100,101]. It has been demonstrated that Scx^+^ tendon cells regulate the initiation of long bone eminences in embryos [102,103]. The conditional knockout of *Bmp4* in Scx^+^ tendon lineage leads to an absence of deltoid tuberosity and several other bone ridges in embryos [102]. Furthermore, the adult Scx null mice showed tendon deficiency and skeletal abnormalities, including the absence of the deltoid tuberosity on the humerus and an underdeveloped patella bone [104]. There are also studies determining the osteogenic role of tendon lineage in trauma or disease states. Achille’s tendon injuries in adult mice led to ectopic cartilage formation derived from Scx^+^ tendon lineage, whereas this was not observed in injured neonatal mice [94]. In addition, the adult Scx^+^ tendon cells also contribute to each stage of the developing heterotopic ossification anlagen and co-express markers of endochondral ossification, such as Osterix and Sox9 [105].

**Figure 3:**
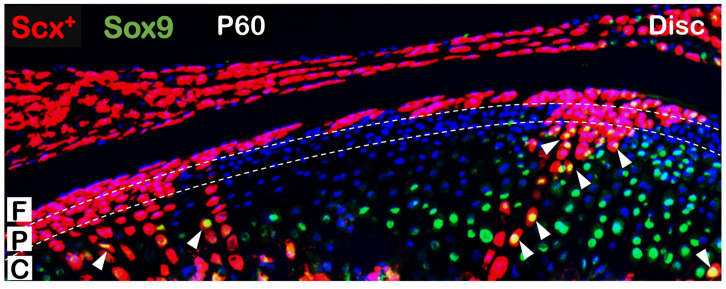
A subset of early labeled Scx^+^ cells contribute to postnatal condyle head expansion. Tamoxifen was injected at P3 and mice were harvested at P60. There were a number of Tomato^+^ cells accumulated into clusters that extended from the fibrous to chondrocytic layers with Sox9 expression (immunofluorescence signal in green color, arrow heads indicating its co-localization with tomato signal) in nuclei. F: fibrous layer; P: prechondroblast layer; C: chondrocytic layer.

Apart from the direct role of tendon lineage to bone formation in normal and disease states, it has also been discovered that tendon cells directly contribute to fibrocartilage formation in mandibular condyles. Ma et al. found that Scx^+^ tendon cells form a subset of chondrocytes in TMJ condyle cartilage via two steps: Scx^+^ tendon cells in fibrous layer first form dendritic prechondroblasts and subsequently transdifferentiate into Scx^+^ chondrocytes [106] (Figure 3). This transdifferentiation was found not only in early postnatal development where tracing started from postnatal day 3, but also in adulthood, where tracing began from postnatal day 28. Interestingly, these tendon-derived chondrocytes do not transdifferentiate into bone cells as the non-tendon-derived chondrocytes do [10,24]. Furthermore, the Scx^+^ derived chondrogenesis, similar to non-Scx^+^ derived chondrocytes [87], is highly sensitive to changes in phosphorus levels as demonstrated in the *Dmp1* KO mice. In addition, the group also stated that the TMJ disc, which is composed of dense fibrocartilagenous tissue, solely originates from tendon lineage.

These observations collectively indicate that tendon cells, beyond their conventional role in skeletal movement, are also directly responsible for the postnatal growth of long bones and joints such as the TMJ condyle. Mature tendon cells are also capable of switching their fates to form ectopic cartilage and bone during trauma and disease states.

## 4. Adipocytes, New Source for Bone and Cartilage Formation?

Adipogenesis is defined as the process by which MSC-derived preadipocytes differentiate into mature adipocytes [107]. Adipogenesis has long been viewed as a unidirectional process, but new data has shown that mature adipocytes can transdifferentiate into osteoblasts through a dedifferentiated stage under certain environmental stimuli [108,109,110,111,112]. The isolated lipid-filled subcutaneous adipocytes from humans lost their lipid content and adopted a spindle-shaped fibroblast-like morphology when cultured in the presence of 10% fetal calf serum (FCS) [108]. After 1–2 weeks, the cell layer consisted mostly of fibroblast-like “dedifferentiated” adipocytes. Subsequently, culturing these dedifferentiated adipocytes in an osteoblast medium enabled them to maintain their fibroblast-like morphology, become positive for alkaline phosphatase (ALP) staining, and express mRNAs for Cbfa1/Runx2, ALP, and osteocalcin. Furthermore, these dedifferentiated adipocytes cultured in osteoblast medium were able to form lamellar bone after being implanted subcutaneously in immunodeficient mice. On the other hand, adipogenic-differentiated cells derived from MSC can also transdifferentiate into chondrogenic-differentiated cells in vitro via dedifferentiation, in correlation with cell cycle arrest [113]. In summary, the isolated and the cultured adipocytes have shown osteogenic and chondrogenic potential in vitro. Future studies using cell lineage tracing may provide direct evidence for adipocytes-to-osteoblasts/chondrocytes transdifferentiation in vivo, which will shed light on developing new solutions for bone pathologies, such as aging and osteoporosis.

## 5. Conclusions and Future Research Direction

By taking advantage of cell lineage tracing and improved imaging methods, the secrets of skeletal development and diseases have been gradually uncovered. Perhaps there is no “terminal” differentiation state for a cell lineage, only a “stable” differentiation state. In the case of HC, the hypertrophic state is just their “terminal” stage within the chondrocyte lineage. However, they take a different developmental trajectory to become bone cells. This is also observed in tendon cells, although their transition to chondrocytes takes multiple steps (from a flat fibrous cell to a round or oval chondrocyte via the irregular and the dendritic pre-chondroblasts) in contrast to the one-step transdifferentiation from chondrocytes to bone cells.

The research on cell transdifferentiation during musculoskeletal development is still in its rudimentary stages, and it poses several unresolved questions. First, what is the molecular mechanism behind these cell transdifferentiation processes? Revealing the key factors involved in the switch of gene expression profiles will provide an effective framework to modulate cell reprograming between cell types. More transcriptomic studies with bulk or single-cell RNA sequence technology [114] will help to unveil the answer. Second, what is the role of the microenvironment during cell transdifferentiation? Either chondrocyte-to-bone cells or tendon-to-chondrocytes during normal development occurs on the boundary between two different tissues. Exploring the effects of environmental stimuli on cell transdifferentiation will provide new avenues to treat various bone diseases and improve fracture healing methodologies. Third, there is still a debate on the model of cell transdifferentiation, whether it is a direct process or intermediate transdifferentiation with a dedifferentiation and redifferentiation stage. For instance, the rapid change in cell fate from chondrocyte to bone cells seems to give little time for the creation of a new stem cell or progenitor cell. However, some studies identified the expression of stem cell-like markers, such as Sox2, Oct4, and Nanog, by chondrocytes during fracture callus formation [67]. This suggests the possibility of a dedifferentiation step in the transdifferentiation process. Notably, proliferating HC were observed in chondro-osseous junctions of the metaphysis and the TMJ condyle [17,19]. Therefore, the model of cell transdifferentiation is still under discussion, and factors such as skeletal site specificity, different developmental stages, and different lineage transitions may be involved in this debate. Finally, translation of the cell transdifferentiation phenomenon to applications for improving human health will be the next challenge to scientists. Due to methodological limitations, it is impossible to apply cell lineage tracing in combination with conditional gene knockout on humans. Single-cell RNA sequencing with human tissues may be a valid alternate approach to screen for the key factors controlling cell fate in normal and disease states in humans. Additional testing with animal models will be required to validate results obtained from human samples in order to accelerate the animal-to-human translation.

## Data Availability

Not applicable.
